# NAD+ metabolism and therapeutic strategies in cardiovascular diseases

**DOI:** 10.1016/j.athplu.2024.06.001

**Published:** 2024-06-11

**Authors:** Chongxu Shi, Zhaozhi Wen, Yihang Yang, Linsheng Shi, Dong Liu

**Affiliations:** a Nantong Laboratory of Development and Diseases, School of Life Science, Nantong University, Nantong, China; b Department of Cardiology, The Second Affiliated Hospital of Nantong University, Nantong, China; c Co-Innovation Center of Neuroregeneration, Key Laboratory of Neuroregeneration of Jiangsu and Ministry of Education, Nantong University, Nantong, China

**Keywords:** Nicotinamide adenine dinucleotide, Atherosclerosis, Cardiovascular diseases, Vascular disorder

## Abstract

Nicotinamide adenine dinucleotide (NAD+) is a central and pleiotropic metabolite involved in cellular energy metabolism, cell signaling, DNA repair, and protein modifications. Cardiovascular diseases (CVDs) are the leading cause of death worldwide. Metabolic stress and aging directly affect the cardiovascular system. Compelling data suggest that NAD + levels decrease with age, obesity, and hypertension, which are all notable risk factors for CVD. In addition, the therapeutic elevation of NAD + levels reduces chronic low-grade inflammation, reactivates autophagy and mitochondrial biogenesis, and enhances oxidative metabolism in vascular cells of humans and rodents with vascular disorders. In preclinical models, NAD + boosting can also expand the health span, prevent metabolic syndrome, and decrease blood pressure. Moreover, NAD + storage by genetic, pharmacological, or natural dietary NAD + -increasing strategies has recently been shown to be effective in improving the pathophysiology of cardiac and vascular health in different animal models, and human health. Here, we review and discuss NAD + -related mechanisms pivotal for vascular health and summarize recent experimental evidence in NAD + research directly related to vascular disease, including atherosclerosis, and coronary artery disease. Finally, we comparatively assess distinct NAD + precursors for their clinical efficacy and the efficiency of NAD + elevation in the treatment of major CVD. These findings may provide ideas for new therapeutic strategies to prevent and treat CVD in the clinic.

## Graphical abstract

**Figure:**
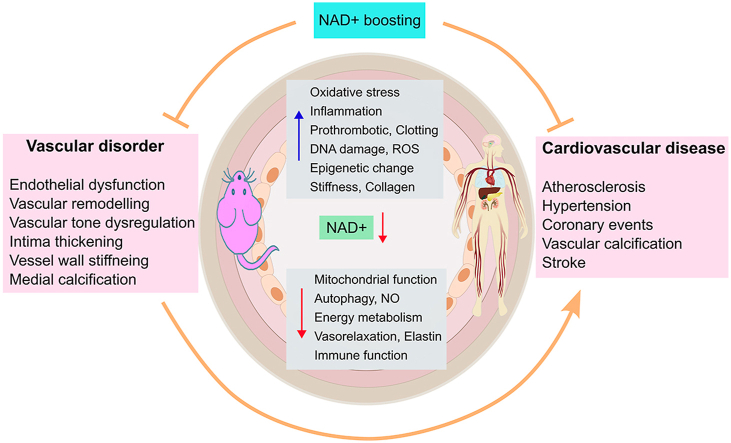

## 1 Introduction

Cardiovascular diseases (CVDs) are the leading cause of death in the elderly and have become a threatening factor for human health and longevity. Vascular dysfunction is a primary risk factor for many CVDs [1]. However, the pathogenesis of many CVDs remains unclear. Thus, to prevent and treat CVDs, it is urgent to unravel the pathophysiological mechanisms of vascular dysfunction. Emerging preclinical studies have indicated that alterations in NAD + levels have a significant impact on cellular metabolism and energetics. Studies have demonstrated that NAD + level alterations in NAD + homeostasis are observed in many diseases, including cancer, CVDs, diabetes, and neurodegenerative and metabolic disorders [[2], [3], [4], [5], [6], [7], [8]].

NAD+ was first discovered in yeast extracts; it is an essential coenzyme for redox reactions and plays a vital role in cellular energetics, metabolism, and mitochondrial functions. In addition to energy metabolism, NAD+ is used as a cofactor or cosubstrate for non-redox NAD + -dependent enzymes; it has multiple roles in many other cellular processes, such as DNA repair, inflammation, intracellular trafficking, aging, and cell death and survival [[2], [3], [4],^9^,^10^].

This review first revises the current understanding of primary NAD + biosynthetic and degradative pathways under healthy conditions. Second, we discuss the vasoprotective mechanisms of NAD+ and the possible consequences of lower NAD + levels on cellular processes which are important to vascular functions. Third, we describe the effect of NAD + levels on the pathogenesis of various CVDs. Finally, we review recent preclinical studies to explore the potential therapeutic effects of boosting NAD + on vascular disorders, including NAD + precursors, and small-molecule drugs that promote NAD + biosynthesis.

## 2 NAD + metabolism in the circulation

In eukaryotic cells, NAD + plays a vital role in regulating energy metabolism in the inner membrane of the mitochondrion [11]. In redox reactions, NAD + can carry electrons (NADH), generating adenosine triphosphate (ATP). During catabolic processes, NAD + can be converted to NADH or phosphorylated to NADP + via NAD + kinases. NAD + also serves as a critical substrate for many enzymes, especially sirtuins (SIRTs), adenosine diphosphate (ADP)–ribose transferases (ARTs) and polymerases (PARPs), and cyclic ADP-ribose (cADPR) synthases (CD38 and CD157) [2]. As important coenzymes for cellular metabolism, NAD+ and NADP + are involved in many cellular processes, such as DNA repair, mitochondrial biogenesis, gene expression, the cell cycle, the cellular stress response, and cellular communication [[12], [13], [14], [15], [16], [17]]. It has been reported that more than 400 proteins are associated with NAD + metabolism [5,[18], [19], [20], [21]].

### 2.1 NAD + biosynthesis

**Fig. 1:**
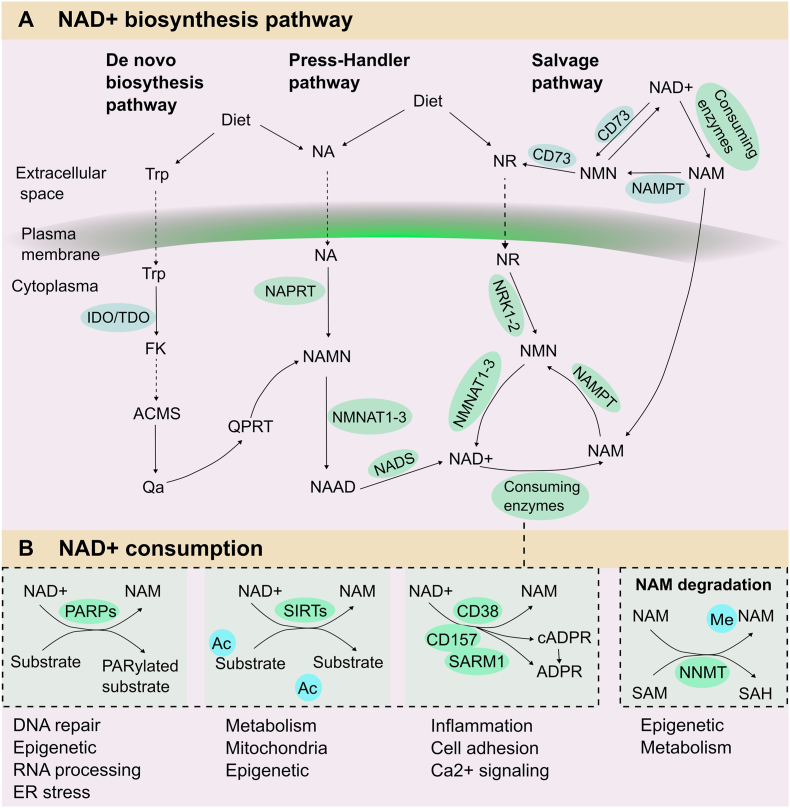
**NAD** + **metabolism in mammals.** NAD + homeostasis is maintained by synthesis, consumption and regeneration in different pathways regulated by specific NAD + -consuming enzymes, generation enzymes and redox reactions. **A: NAD** + **biosynthetic pathway**. Three independent pathways maintain NAD + levels. The dietary amino acid Trp is converted to NAD + via a de novo synthesis pathway. After Trp enters the cytoplasm, the rate-limiting enzyme (IDO/TDO) will transfer Trp to FK. Following several steps, the ACMS was generated and condensed into Qa spontaneously; subsequently, Qa was converted to QPRT to produce NAMN, which converges with the Preiss-Handler pathway. The Press-Handler pathway uses NA as the original material. NA can be converted to NAMN via the NAPRT enzyme, followed by NAAD generation via NMNAT enzymes; in the end, NAD+ is generated via NADS synthetase. The salvage pathway mainly involves recycling the byproduct NAM generated during NAD + consumption. In the cytoplasm, NAMPT converts NAM to NMN, one of the NAD + precursors, and NMNATs will convert NMN into NAD+. In the extracellular space, NAM is transformed into NMN first, and NMN is then dephosphorylated by CD73 to NR. NR is converted to NMN via NRK enzymes in the intracellular space. Ultimately, NAD+ is generated via NMNATs. **B: NAD** + **consumption**. NAD + acts as a cosubstrate for a wide variety of enzymes, including PARPs, sirtuins, CD38/CD157, and SARM1. These enzymes use NAD + as a cosubstrate to modulate various biological processes, generating their byproduct, i.e. NAM. These enzymes have an impact on DNA repair, RNA processing, metabolism, genomic stability, inflammation, cell adhesion, and stress resistance. *Abbreviations:* NAD+, nicotinamide adenine dinucleotide; IDOs, indoleamine 2,3-dioxygenase; TDO, tryptophan 2,3-dioxygenase; QA, quinolinic acid; NAMN, nicotinate mononucleotide; QPRT, quinolinate phosphoribosyl-transferase; NAPRT, nicotinic acid phosphoribosyltransferase; NMNATs, nicotinamide mononucleotide adenylyl transferases; NR, nicotinamide riboside; Trp, tryptophan; PARPs, poly (ADP-ribose) polymerases; NNMT, nicotinamide N-methyltransferase; NMN, nicotinamide mononucleotide; NAM, nicotinamide; NA, nicotinic acid. NAMPT, Nicotinamide phosphoribosyltransferase.

*The salvage pathway:* NAD + biosynthesis is modulated by three different pathways in organisms: the de novo biosynthesis pathway, the Preiss–Handler pathway, and the salvage pathway (Fig. 1A). The salvage pathway is the main synthesis mode by reusing nicotinamide (NAM) generated as a byproduct of NAD + consumption. Several biosynthetic enzymes, including SIRTs, ARTs, and PARP, can convert NAD + to NAM, which acts as the NAD + precursor. Following that the rate-limiting enzyme nicotinamide phosphoribosyltransferase (NAMPT) will convert NAM into mononucleotide (NMN). On the one hand, NMN is converted into NAD + by mononucleotide adenylyltransferase (NMNAT) in the cytoplasm [22]. On the other hand, NMN can also be transferred to nicotinamide riboside (NR) by CD73 in the extracellular space, and then the intracellular NR will be transferred to NMN; this process is regulated by nicotinamide ribokinase (NRK) 1 or NRK2 [23]. Moreover, NR can also be taken from a regular diet.

*The* de novo *pathway:* The de novo synthesis pathway mainly depends on tryptophan (Trp), which can only be obtained from the diet. Two critical steps participate in this pathway. The first step is to transform Trp into N-formylkinurenine (FK) through indoleamine 2,3-dioxygenase (IDO) or tryptophan 2,3-dioxygenase (TDO). These two enzymes can also regulate the activity of the immune response and reproductive and central nervous systems in mammals. After several complicated steps, FK is converted into 2-amino-3-carboxylic acid salt semialdehyde (ACMS). Then, ACMS spontaneously condenses to quinoline acid (Qa), and Qa is converted into nicotinic acid mononucleotide (NAMN). Afterward, the transformation of NAMN is linked with the Preiss-Handler pathway to generate NAD + [2].

*The Preiss-Handler synthesis pathway:* NA, mainly obtained from food, is another main precursor of NAD+. In the Preiss-Handler synthesis pathway, NA from the diet is converted to NAMN through NA phosphate ribosyltransferase (NAPRT). Then, the NAMN derived from NA and Trp are converted to nicotinate adenine dinucleotide (NAAD) by NAMN transferase (NMNAT). NAAD can be directly converted into NAD + via NAD + synthase (NADS) [24]. Hence, NMNAT is a key enzyme in this pathway.

### 2.2 NAD + consumption

NAD+ is a key substrate of many enzymes involved in various biological processes (Fig. 1B). During the glycolysis process, NAD+ is reduced to NADH, and the oxidation of NADH causes high-energy electrons to transfer to the electron transport chain in the mitochondrion, generating proton dynamics and ATP. NAD + can also be phosphorylated to NADP+, and NADP+ and its degraded form NADPH participate in many important redox reactions. NADP+ and NADPH act as the cofactors of glutathione reductase, protecting cells from oxidative stress. In addition, NADPH is also related to the synthesis process of sugars and nucleic acids [25].

*Sirtuins*: NAD + can be cleaved by SIRTs that use NAD + as a cosubstrate. There are seven members (SIRT1-7) in the SIRT family in mammals, and they are expressed in different cellular compartments. SIRT1, SIRT6, and SIRT7 are located in the nucleus, SIRT2 in the cytoplasm, and SIRT3-5 in the mitochondria. SIRTs are energy sensors, they can regulate cellular metabolism and are responsible for approximately 1/3 of total NAD + consumption under steady conditions [26]. Recently, evidence has indicated that nuclear SIRT1, SIRT6, and SIRT7 regulate DNA repair and genome stability, and both mitochondrial SIRT3-5 and nuclear SIRT1 regulate mitochondrial homeostasis and metabolism [27]. Depending on NAD+, SIRTs catalyze the removal of acyl units from lysine residues on proteins, generating NAM and ADP-ribose. Adults who have low SIRT1 levels tend to develop premature microvascular dysfunction, and they may also have a higher risk of developing CVD [28]. Consistently, animal experiments confirmed that *Sirt1* knockout mice display marked endothelial dysfunction that enhances the progression of micro- and macrovascular complications [29].

*PARPs*: The PARP family consists of 17 proteins in humans, but only PARP1, PARP2, and PARP3 are located in the nucleus; they respond to DNA damage in the early phase and improve DNA damage repair [[30], [31], [32]]. This process consumes massive amounts of NAD+ and is widely related to the pathological process. Generally, NAM and ADP-ribose are the byproducts of PARP-mediated NAD + cleavage, and the cleavage process also produces ribosyl-ribosyl complexes representing a signal for other DNA-repairing enzymes [33,34]. Notably, uncontrollable DNA damage results in the overactivation of PARPs leading to the depletion of NAD+. This process impairs the glycolysis rate, mitochondrial electron transport chain, and ATP production, ultimately triggering endothelial cell death [35,36]. In mice fed a high-fat diet (HFD), PARP depletion groups had enhanced NAD + levels, increased SIRT1 activity, and improved mitochondrial function [37,38]. Similar effects were also observed in patients. Overall, targeting PARPs, particularly, PARP1, is a promising therapeutic strategy to rebalance NAD + levels in the CVD.

*cADPR*: Several ADPR members also functionally depend on NAD+. CD38 and CD157 are multifunctional ectoenzymes characterized by the glycohydrolase and ADP-ribosyl cyclase activities. ADPR splits the glycosidic connection within NAD + to generate NAM and ADP-ribose, whereas ADP-ribosyl cyclase activity generates cyclic ADP-ribose. CD38 executes a base-exchange reaction in acidic conditions; it can trade the NAM of NAD(P) + for NA and generate nicotinic acid adenine dinucleotide (phosphate) (NAAD(P)) [39]. Notably, CD38 employs NMN as an alternative substrate [40,41], while CD157 applies NR as an alternative substrate [42,43]. Thus, small-molecule inhibitors targeting CD38 and CD157 may be considered NAD + precursor metabolites to restore NAD + levels in aging individuals.

## 3 NAD+ and vascular function

**Fig. 2:**
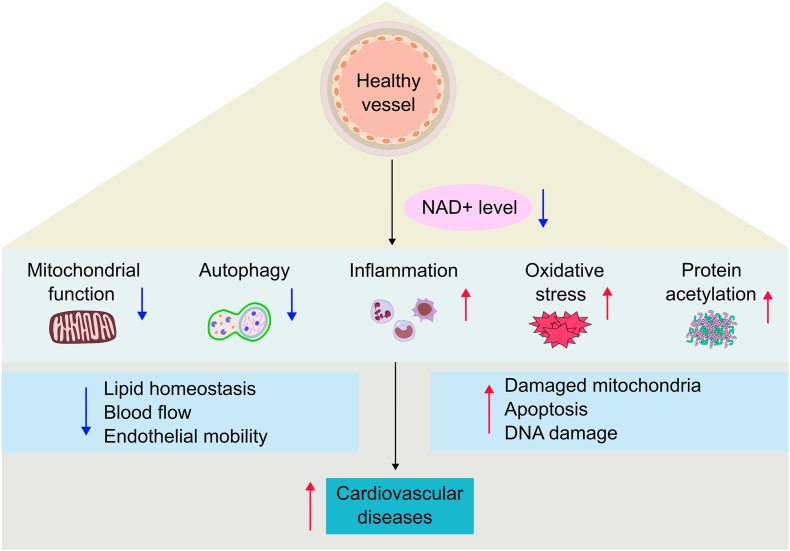
**NAD** + **in vascular health and function.** NAD + has multiple roles in maintaining vascular health. On the one hand, decreased NAD + levels reduce mitochondrial function and autophagy, leading to increased numbers of damaged mitochondria, apoptotic cells and damaged DNA. On the other hand, suppressed NAD + levels also enhance the inflammatory response, oxidative stress and protein acetylation, which lower blood flow and restrain endothelial mobility and lipid homeostasis. Together, this furthers the prevalence of CVD. *Abbreviations:* NAD+, nicotinamide adenine dinucleotide; CVD, cardiovascular disease.

Vascular health is fundamentally important for homeostasis. The endothelium plays multiple roles in maintaining homeostasis, such as blood filtration, vessel tone adjustment, immune response modulation, hormone trafficking, and angiogenesis [44]. The alterations of vascular structure and function lead to a vasoconstrictive, prothrombotic, and proinflammatory state and extracellular matrix changes (Fig. 2). Ultimately, CVD development [45].

### 3.1 Oxidation and mitochondrial stress

Experimental studies confirm that oxidative stress is mechanistically associated with cardiac and vascular diseases and also contributes to disease progression [45]. Under physical conditions, the intracellular levels of reactive oxygen species (ROS) are controlled by an intricate array of antioxidant defense systems and the peroxiredoxin/thioredoxin system, and the imbalance between these systems contributes to oxidative stress. Under pathological conditions, ROS products, like superoxide anion (O^2−^) and the nitric oxide radical (NO), are highly produced. The elevation of ROS overwhelms cellular antioxidant defenses and diminishes ROS scavenging, which results in cardiac dysfunction [[46], [47], [48], [49], [50]]. The aged vascular system produces large amounts of ROS, superoxide, and hydrogen peroxide. These products impair vascular dilation activity and promote the formation of the toxic free radical peroxynitrite. Mitochondrial function in endothelial cells (ECs) highly depends on the intracellular level of NAD + since intracellular NAD + levels have an effect on fatty acid β-oxidation and oxidative phosphorylation in mitochondria. The accumulation of intracellular ROS is considered as a driving factor for mitochondrial dysfunction [51]. Research has shown that supplementation with NAD + precursors can attenuate vascular oxidative stress and improve mitochondrial and vascular dysfunction [52,53]. Similarly, administration of NMN can reduce superoxide production in aged mice, thereby reversing age-related oxidative stress in arteries [52] and improving mitochondrial membrane potential and mitochondrial function [54]. Additionally, NMN administration also shows a protective effect on neurovascular function by activating mitochondrial bioenergetics in mice [55]. Furthermore, upregulation of SIRT1 can also improve mitochondrial function by attenuating ROS production and activating the antioxidant defense system in mice [56,57]. At the same time, NMN also reduces vascular oxidative stress by deacetylating many mitochondrial proteins (e.g., superoxide dismutase 2, SOD2) in a SIRT3-dependent manner [58]. NMN was able to revert changes in the expression of the microRNA profile that correlated with enhanced mitochondrial biogenesis in mouse aorta [59].

Another approach to restoring intracellular NAD + levels is to block NAD + -consuming enzymes [3], such as cyclic ADP-ribose synthase CD38, which is highly expressed in the endothelium [60,61]. The expression of CD38 is strongly activated by hypoxia-reoxygenation, leading to loss of eNOS-mediated NO generation and exaggerated eNOS uncoupling. Hence, the depletion of NAD + might markedly affect mitochondrial redox balance with implications for vascular disease risk.

### 3.2 Inflammation

Inflammageing refers to chronic systemic low-grade inflammation, represents a hallmark of aging, and is tightly related to the development of CVD [62,63]. Chronic inflammation has effects on systemic metabolic processes, such as glucose and lipid uptake, and insulin sensitivity, via intricate crosstalk between immune cells and metabolism. Notably, persistent low-grade inflammation has a causal link to an age-dependent decrease in NAD + [64]. CD38 is a multifunctional enzyme and is considered one of the principal regulators of cellular NAD + levels in mammals. A recent study found that senescent cells can promote the proliferation of proinflammatory mouse macrophages, and these inflamed macrophages express a high level of CD38 in aged mice. Consistently, CD38-overexpressing macrophage aggregation in liver and adipose tissue results in age-dependent NAD + reduction [64]. CD38 is highly expressed in endothelial cells [61], but upregulation has been observed in human macrophages and monocytes in inflammatory conditions [65], as well as in blood samples from aged individuals [66]. Therefore, age-induced inflammatory processes could be attenuated by fighting NAD + consumption, increasing NAD + synthesis. NAM is the precursor of NAD+, and a study demonstrated that administration of NAM can boost NAD + synthesis and strongly reduce the proinflammatory phenotype in mice [67]. Similarly, chronic supplementation of NAM in aged mice fed an HFD can induce a marked reduction in inflammation and ameliorate healthspan [68]. Niacin, another NAD + precursor, had similar anti-inflammatory actions and promoted cardiac healing after myocardial infarction in mice [69].

Both NMN and NR are alternative NAD + precursors, and these molecules display similar anti-inflammatory effects as NAM. An *ex vivo* study showed that NMN and NR can suppress interleukin-1β (IL-1β) and tumor necrosis factor-α (TNF-α)-induced inflammation in ECs [70]. Moreover, NMN and NR administration can also ameliorate endothelium-dependent vasodilation in murine aortic rings [70]. Interestingly, NMN revoked endothelial dysfunction and inflammation by extracellular conversion to NR through CD73, although the vasoprotective effects upon NR were not related to CD73 [70]. CD73 is an enzyme present on the luminal surface of the endothelium. Thus, strategies to enhance vascular NAD + levels might be a promising approach to prevent inflammatory-mediated endothelial dysfunction and consequent vascular diseases.

### 3.3 Autophagy

Autophagy is a dynamic and reparative intracellular process. Autophagy can reduce oxidative stress associated with superoxide, enhance the bioavailability of NO, and exert anti-inflammatory effects on arteries; therefore, autophagy plays a significant role in maintaining vascular endothelial function. Defective autophagic flux is a common cause of vascular dysfunction and the development of vascular diseases. Reducing the expression of autophagy genes in the vascular endothelium of mice or completely blocking autophagy significantly worsens vascular physiology [71]. Accordingly, a study showed that loss of *Atg 7* (involved in autophagosome formation) in smooth muscle cells (SMCs) leads to abnormal vascular reactivity and reduced contractility of SMCs [72]. These findings suggested that an intact autophagic response is important to maintain the normal homeostasis of blood vessels. Enhancing autophagy can improve endothelial dysfunction in blood vessels [73]. Therefore, increasing intracellular NAD + levels can enhance autophagy to alleviate vascular diseases. For instance, NAD + supplementation can restore autophagy to reduce microvascular damage, maintaining microvascular density and integrity in rat hearts [74]. Notably, studies have demonstrated that sirtuins are important in regulating NAD + -induced autophagy. SIRT1 deacetylase is known as an important regulatory factor in autophagy [75].

## 4 NAD+ in cardiovascular diseases

**Fig. 3:**
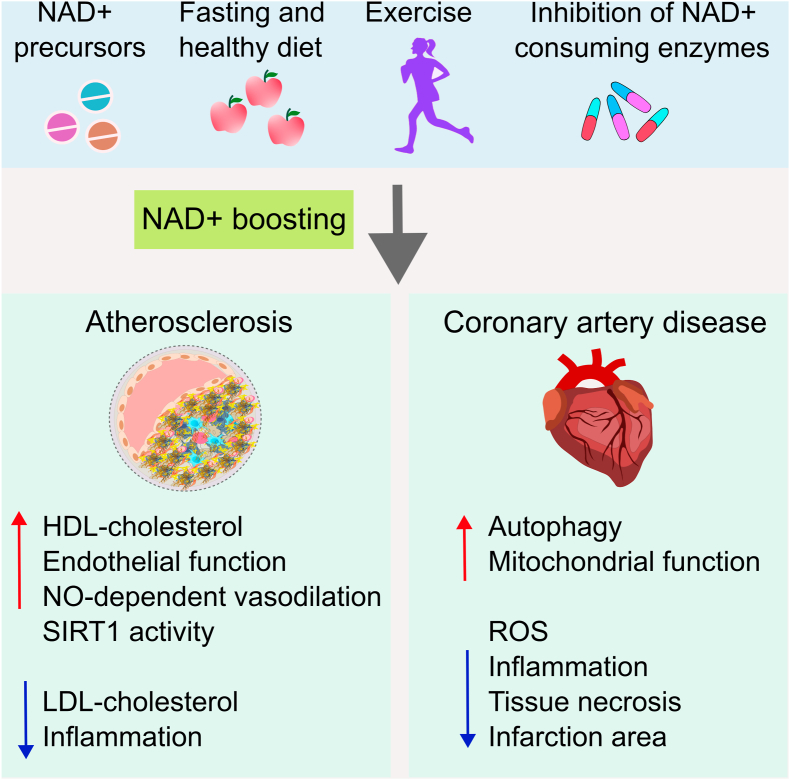
**Therapeutic approaches to restore NAD** + **levels and their impact on CVD**. Several strategies can boost NAD + levels, including supplementation with NAD + precursors, inhibition of NAD + -consuming enzymes via pharmacy, fasting or taking a healthy diet, and increased exercise and NAD + -boosting strategies can develop better CVD outcomes. In Atherosclerosis, high NAD + levels can reduce chronic inflammation and decrease LDL-cholesterol levels. It can also increase endothelial function and vasodilation. In coronary disease, elevation of NAD + levels can increase autophagy and mitochondrial function and lessen ROS release, inflammation, and tissue necrosis. *Abbreviations:* NAD+, nicotinamide adenine dinucleotide; CVD, cardiovascular disease; HDL, high-density lipoprotein, LDL, low-density lipoprotein; NO, nitric oxide; ROS, reactive oxygen species.

Low-grade chronic inflammation is the basic trigger of vascular dysfunction and related diseases, and a systemic decline in NAD+ is associated with inflammation. Importantly, imbalanced NAD + metabolism has also been observed in vascular pathologies, including atherosclerosis and coronary artery diseases (Fig. 3). Therefore, pharmacological restoration of NAD + homeostasis therapies has been applied to ameliorate CVD in animal models (Table 1).

**Table 1:** Experimental studies of NAD + -boosting strategies in CVD.

Vascular disease	Experiment design	Vascular-related outcomes	Ref.
**Atherosclerosis**	Supplementing NAM in *ApoE−/−* mice.	Prevented the development of atherosclerosis.	71
	Specific knockdown of *ApoE−/−* mouse.	Reduced the area of arterial plaques, the number of macrophages and cell apoptosis.	75
**Acute myocardial infarction**	NAD + precursors administration in I/R rats.	Reduced I/R induced myocardial infarction.	83
	Supplementing NAD + precursor after knocking down CDR1as in MI mice.	Reduced arrhythmia caused by AMI.	84
	Overexpression of NAMPT in ischemia-reperfusion mice.	Reduced myocardial infarction area and myocardial cell apoptosis.	85
**Acute cerebral infarction**	NAD + exogenous administration in I/R mice.	Reduced infarct size, edema formation, and neurological deficits.	78
**Coronary artery disease**	Pig myocardial IRI model with external aid NAD + administration.	Reduced myocardial necrosis and promote cardiac function recovery.	86
	Overexpressing SUR 2A mice or mice on a nicotinamide rich diet.	Increased the resistance of the heart to I/R.	87
	Melatonin post-treatment and NMN on elderly rats.	Reduced oxidative stress and mitochondrial ROS levels.	88

*Abbreviations:* NAD+, nicotinamide adenine dinucleotide; NAM, nicotinamide; NAMPT, Nicotinamide phosphoribosyltransferase; NMN, nicotinamide mononucleotide; NAM, nicotinamide; ROS, reactive oxygen species; AMI, acute myocardial infarction.

### 4.1 Atherosclerosis

Atherosclerosis is a chronic inflammatory vascular disease, and endothelium activation by overloaded cholesterol is the initial step, followed by an inflammatory response by recruiting M1-like macrophages and a decline in SMCs in the vasculature [[76], [77], [78]]. Several signaling pathways are involved in the inflammatory response during the development and regression of atherosclerosis, such as NLRP3 inflammasome, TLR, and SIRTs. For example, SIRT1 regulates cholesterol biosynthesis in the liver, leading to a decreased level of serum lipids [79]. Fatty acid, cholesterol, and glucose metabolism are regulated by the liver X receptor (LXR) protein, and SIRT1 can promote LXR deacetylation in an NAD + -dependent manner. Moreover, SIRT1 inhibits foam cell formation [80]. Of note, administration of NAM in *ApoE*−/− mice can prevent atherogenesis and suppress lipoprotein oxidation and aortic inflammation [81]. A double-blind, randomized, placebo-controlled study has indicated that methyl-NAM can promote vasodilation in a NO-dependent manner, thereby executing anti-thrombotic and anti-inflammatory effects and furthering improved endothelial function [82]. Similarly, in *ApoE-* and *Ldlr-*deficient mice, methyl-NAM showed atheroprotective effects by reducing atherosclerotic plaque area, plaque inflammation, and cholesterol content in the brachiocephalic artery [83]. In *ApoE*−/− mice, methyl-NAM had similar protective effects on endothelium-dependent vasorelaxation [84].

NAMPT, the rate-limiting enzyme of NAD + salvage biosynthesis, is considered to be associated with atherosclerosis. Studies have found that systemic knockdown of *Nampt* showed an atheroprotective effect in *ApoE*−/− mice, and these mice had reduced plaque area, macrophage numbers, and cell apoptosis [85]. Overexpression of NAMPT in leukocytes specifically attenuated atherosclerotic plaques in *Ldlr*−/− mice [86]. In contrast, global NAMPT overexpression aggravated atherosclerosis in *ApoE*−/− mice [87]. Atherosclerosis has many complications, and NAD + can significantly reduce ischemic brain injury by preventing autophagy [88]. In a cerebral artery occlusion model, administration of NAD + before reperfusion significantly reduced infarct size, edema formation, and neurological deficits after 48 h of ischemia. NAD+ is also a substrate for PARP, a poly (ADP-ribose) polymerase. After DNA damage, activated PARP leads to the deletion of NAD+ and ATP [89]. Animal studies have shown that PARP pharmacological inhibition and gene deletion both reduced plaque sizes in *Apoe−/−* mice concomitant with reduced macrophage homing [90,91]. Moreover, pharmacological inhibition of PARP activity displayed antiatherogenic effects and reduced atherosclerotic plaque in the atherosclerosis mouse model [92]. Notably, a recent report confirmed that PARP improved endothelial function by preserving NAD + levels in rabbits [93].

### 4.2 Coronary artery disease

Coronary artery diseases (CAD) are the major consequence of atherosclerosis, and acute myocardial infarction (AMI) is a common event of CAD and is associated with NAD + deficiency [[94], [95], [96]]. The reduction in NAD+ in postischemic hearts is due to the high activity of CD38 in ECs [61]. CD38 overactivation may be a potential cause of postischemic endothelial dysfunction, implying that CD38 is a possible target for preventing endothelial dysfunction in CAD [97]. Evidence has shown that the administration of NAD + reduces apoptosis and the infarction area in the heart in a dose-dependent manner [98]. The mRNA expression of *CDR1as* showed a significant difference between AIM patients and healthy subjects, which indicated that *CDR1as* mRNA in serum might be a potential biomarker for AMI detection [99]. Moreover, CDR1as can cause NAD + depletion and mitochondrial dysfunction by directly inhibiting NAMPT expression, and NAD + supplementation can alleviate CDR1as levels after AMI [100]. Cardiac-specific overexpression of NAMPT increased NAD + levels and reduced AMI size and cardiomyocyte apoptosis after ischemia/reperfusion (I/R) [101].

In a pig AMI model, NAD + administration significantly reduced cardiomyocyte necrosis, enhanced glucose metabolism, and promoted cardiac function recovery [102]. NAD + can also reduce inflammation and cardiac fibrosis and improve ventricular compliance [102]. The NR-enriched diet can upregulate SUR2A expression and significantly reduce the area of AMI after I/R in mice [103]. NMN administration also has significant protective effects on AMI in elderly rats. NMN can reduce oxidative stress and mitochondrial ROS levels and increase mitochondrial membrane potential, restoring the NAD+/NADH ratio [104].

In addition, pharmacological and genetic CD38 inhibition to increase cellular NAD + dramatically suppressed angiotensin II-induced hypertension and vascular remodeling in mice [105]. Mice with downregulated CD38 levels displayed lower blood pressures, reduced vascular media thickness, media-to-lumen ratio, collagen deposition, and normalized elastin expression. Moreover, NMN supplementation and CD38 inhibition alleviated the senescence of vascular SMCs [105].

## 5 NAD + as a target to improve vascular health: from bench to bed

Epidemiological and preclinical studies found that intracellular NAD + levels decrease in various tissues and species, including humans [41,64,[106], [107], [108], [109], [110], [111]]. NAD + level is an important factor in maintaining health conditions, including in the cardiovascular system [112,113]. Therefore, clinical and preclinical studies in restoring NAD + metabolism are growing (Table 2), and restoring NAD + offers exciting new biological insights and therapeutic opportunities for CVD patients.

**Table 2:** Clinal trials focusing on NAD + restoration.

Application of substances	Time frame	Condition of participants	No. of recruited participants	Vascular-Related Outcomes	Ref.
**Resveratrol**	24 months	Postmenopausal women aged 45 to 85.	146	Improvement of cerebrovascular function.	ACTRN12616000679482p
	30 days	Obese patients.	150	Energy metabolism and changes in systolic blood pressure.	123
**MNM**	60 days	Healthy subjects between the ages of 40 and 65.	66	Changes in serum NAD+/NADH levels and blood pressure.	NCT04228640
	28 days	Healthy volunteers aged 30 to 60.	20	Changes in arterial blood pressure, heart rate, and blood lipids.	NCT04862338
**NR**	6 weeks	Healthy middle-aged people.	30	Changes in blood pressure.	NCT02921659
	6 weeks	Elderly people with hypertension.	49	Changes in systolic blood pressure and arterial stiffness.	NCT04112043
	3 months	Patients with moderate to severe chronic kidney disease.	118	Changes in aortic stiffness and arterial blood pressure.	NCT04040959
**NAM**	48 h	Women with early-onset preeclampsia.	25	Changes in average blood pressure.	NCT03419364

*Abbreviations:* NMN, nicotinamide mononucleotide; NAM, nicotinamide; NR, nicotinamide riboside.

### 5.1 Resveratrol

Several compounds have been used to regulate NAD + levels pharmacologically, and resveratrol, which is extracted from Veratrum grandiflorum, is the most effective. Resveratrol is known as a scavenger of ROS radicals with antioxidative properties [114,115] and is also recognized for anticancer effects [[116], [117], [118]] in lower organisms [119,120]. It can also extend the lifespan of yeast and worms [121,122]. Animal experiments found that compared to mice fed only an HFD, resveratrol administration indicated protective effects in physical conditions, such as lower body weight, improved glucose metabolism, and reduced pancreas and heart injury. Importantly, the protective effect of resveratrol is related to increased mitochondrial quantity and function induced by the activity of AMPK and PGC-1α [123,124]. In the regular diet group, resveratrol also significantly reduced the inflammatory response and apoptotic events in the vascular endothelium [[123], [124], [125]]. However, the mechanism underlying the resveratrol effect is still under debate. Some investigations have suggested that resveratrol activates AMPK first and then indirectly activates SIRT1 by increasing intracellular NAD + levels, thereby improving vascular status [126,127]. Others have proposed that resveratrol may activate SIRT1 first, subsequently deacetylate, and stimulate AMPK kinase to initiate AMPK activation, ultimately enhancing the intracellular levels of NAD + [[128], [129], [130]]. Notably, the mechanism of resveratrol function is dose-dependent [131]. Resveratrol is a nonspecific compound that can interact with many proteins within cells [132]. Hence, elucidating the mechanisms of resveratrol in vascular and related disease protection remains challenging.

Several specific clinical trials have demonstrated that resveratrol can extend lifespan, while its environment-related effects are still under debate [120]. In a randomized double-blind study, healthy obese men received either resveratrol (150 mg/day) or a placebo for 30 days, and resveratrol therapy significantly reduced the resting metabolic rate and blood pressure. Moreover, resveratrol activated AMPK, increased SIRT1 and PGC1α protein expression, and increased mitochondrial activity in muscle cells [133]. However, another trial with nonobese men failed to observe any measurable physiological improvements after 4 weeks of resveratrol therapy [134]. Others also found that resveratrol was able to improve biological parameters of kidney function in patients [135] and improve arterial function by reducing media thickness, inflammation, fibrosis, and oxidative stress and lowering NADH oxidase [136,137].

### 5.2 NAD + precursors

Numerous studies have shown that dietary supplementation of NAD + precursors, such as tryptophan, NA, NAM, NR, and NMN, can effectively increase NAD + levels in animals and humans [4,52].

#### 5.2.1 Nicotinamide riboside (NR)

NR was the first one to be used in clinical trials to evaluate pharmacokinetics in humans [138]. The oral administration of NR is well tolerated with no side effects and increases blood NAD + levels in a dose-dependent manner in clinical trials [139,140]. In heart failure patients, NR administration orally suppressed proinflammatory activation of immune cells by approximately 20 % and improved mitochondrial adaptation [141]. A randomized double-blind chronic supplementation with NR administration observed similar results [142]. In this study, NR supplementation was conducted for 6 weeks in healthy middle-aged and older people, and found NR reduced the stage I hypertension range and aortic stiffness compared with placebo control [142]. These findings imply that NR may reduce the incidence of cardiovascular events. However, not all studies support the therapeutic potential of NR supplementation [143]. NR administration failed to improve endothelial dysfunction in middle-aged and older individuals [138]. Moreover, oral NR supplementation did not improve blood flow, mitochondrial bioenergetics, or skeletal muscle metabolism, although it reduced plasma levels of inflammatory cytokines in 70- to 80-year-old men [144]. To evaluate the efficiency of NR, a phase II clinical trial has already been authorized for heart failure patients (Project-ANR-17-CE17-0015). To assess the safety and tolerability of NR, another interventional clinical trial including 30 patients with systolic heart failure is ongoing [145], but there are no results yet. To reveal the effects of NR on myocardial NAD + levels, mitochondrial function, and inflammatory responses, a study recruited subjects with left ventricular assist device surgery (NCT04528004). More clinical trials are in progress to reveal the therapeutic effects of NR (NCT03151239, NCT03432871, NCT03501433, NCT02835664).

#### 5.2.2 Niacinamide (NAM)

NAM may have no marked effect on atherosclerosis patients since it cannot decisively reduce plasma lipid levels [146]. In contrast, NAM possesses antioxidant and anti-inflammatory capabilities [68,81]. Therefore, NAM may also have benefits for myocarditis. Normally, heart inflammation is caused by a virus and displays infection-related mechanisms and similar signs as other inflamed hearts (cardiac amyloidosis and hypertrophic cardiomyopathy) [[147], [148], [149]]. Moreover, NAD + depletion is tightly related to the pathogenesis of inflamed hearts. This evidence suggests that the administration of NAD + precursors may be a promising therapy for viral myocarditis. Myocarditis was observed in SARS-CoV-2-infected subjects [150]. Interestingly, NAD + -consuming enzymes (SIRT1 and PARP) are stimulated and directly engaged in the activation of intracellular immune responses upon SARS-CoV-2 infection. Consequently, the activation of SIRT1 and PARP may secondarily induce NAD + depletion. Consistently, in SARS-CoV-2-infected patients, vitamin B3 intermediates may be useful in bolstering innate and adaptive immune responses [[151], [152], [153]]. SARS-CoV-2 infection induces adverse diseases in other systems other than the respiratory system. In particular, NAM treatment can effectively reduce the side effects of COVID-19 syndrome [154]. This evidence suggests that NAM or any other NAD + precursor may have a positive effect on preventing this inimical cardiac outcome of COVID-19. Another trial is currently ongoing to evaluate the influence of NAM on myocardial injury in patients undergoing on-pump cardiac surgery (NCT04750616).

#### 5.2.3 Nicotinamide mononucleotide (NMN)

Oral supplementation with NMN can effectively increase NAD + levels and improve various physiological functions which has already been proven in animal models [5,155]. Moreover, administration of NMN for 6 weeks combined with amateur exercise enhanced the ventilatory threshold in middle-aged unprofessional runners, which indicated that NMN may augment oxygen consumption of skeletal muscle rather than cardiac muscle [156]. Although this study did not analyze cardiac function, a recent study concluded that exercise training can increase lipotoxic cardiomyopathy achieved by an HFD in aged Drosophila [157]. Therefore, we hypothesize that NMN administration may further reinforce exercise resistance to HFD-induced cardiomyopathy. To investigate the safety of a single oral administration of NMN, a clinical trial including 10 healthy men of 40–60 years of age was carried out in Japan [158]. They found NMN had no significant effects on heart rate, blood pressure, oxygen saturation, and body temperature [158]. The study suggests that NAD + levels in the body can be safely elevated by NMN. To confirm this hypothesis, further investigations are needed to directly address the potential positive influence of NMN on heart health in future clinical trials.

## 6 Conclusions and future perspectives

In conclusion, targeting NAD + metabolism exhibits meaningful therapeutic potential in managing age-related CVD patients. Indeed, CVD is the major cause of morbidity in the elderly and leads to about 30 % of deaths globally. Thus, improving cardiovascular health would largely reduce the morbidity of CVD, especially in elderly individuals. In the context of vascular diseases, NAD + -boosting strategies may ensure proper vascular health and enhance cardiovascular and cerebrovascular conditions in elderly individuals. To maintain the cellular homeostasis of NAD+ and improve health, NAD + precursors become potential candidates to enhance NAD + levels *in vivo* as NAD + itself is not given to humans directly. Additionally, the use of NAD + precursors combined with small molecules to restore intracellular NAD + levels is a promising therapeutic approach to treat CVD and increase human healthspan. Furthermore, many preclinical studies have focused on transporters and receptors that are involved in NAD + precursor metabolism, and these studies will help us to better understand the molecular mechanisms involved in the NAD + biological process during CVD. To date, the animal and human models for CVD studies have demonstrated that NAD + plays a central role in cellular metabolic homeostasis and healthy living. Currently, several human clinical trials are ongoing to evaluate the safety and efficacy of NAD+. Importantly, early-phase trials of short-term NR/NMN administration have proven that it is safe to increase NAD + levels in healthy participants. However, the side effects of long-term supplementation with NAD + precursors are still unknown, despite several promising preliminary trials. Furthermore, NAD + can modulate vascular inflammation and autophagy, and these processes might be investigated within the broad physiological effects of NAD + precursors *in vivo*. Many other questions need to be explored to expand our understanding of the potential of NAD + -boosting therapies. In the future, to develop NAD + targeting interventions, the mechanisms underlying the action of NAD+ in a cell type- and precursor-specific manner should be elucidated.

## Author contributions

CS drafted the outline of the manuscript, and all authors wrote parts of it, edited the complete version, and approved the final version.

## Declaration of competing interest

The authors declare that they have no known competing financial interests or personal relationships that could have appeared to influence the work reported in this paper.

## Abbreviations

NAD+: nicotinamide adenine dinucleotide;
CVD: cardiovascular disease
ATP: adenosine triphosphate
ADP: adenosine diphosphate
SIRTs: sirtuins
ARTs: ADP-ribose transferases
NAM: nicotinamide;
NMN: mononucleotide;
NAMPT: nicotinamide phosphoribosyltransferase
NR: nicotinamide riboside;
NRK: nicotinamide ribokinase
NA: niacin acid
NAMN: nicotinic acid mononucleotide;
NAAD: nicotinate adenine dinucleotide;
ROS: reactive oxygen species
NO: nitric oxide;
EC: endothelial cell
IL-1β: interleukin-1β
TNF-α: tumor necrosis factor-α;
SMC: smooth muscle cell
CAD: coronary artery diseases
AMI: acute myocardial infarction
I/R: ischemia/reperfusion

## References

[1] Chung M.K., Eckhardt L.L., Chen L.Y., Ahmed H.M., Gopinathannair R., Joglar J.A., Noseworthy P.A., Pack Q.R., Sanders P., Trulock K.M. (2020). Lifestyle and risk factor modification for reduction of atrial fibrillation: a scientific statement from the American heart association. Circulation.

[2] Verdin E. (2015). NAD^+^ in aging, metabolism, and neurodegeneration. Science.

[3] Abdellatif M., Sedej S., Kroemer G. (2021). NAD(+) metabolism in cardiac health, aging, and disease. Circulation.

[4] Yoshino J., Baur J.A., Imai S.-I. (2018). NAD(+) intermediates: the biology and therapeutic potential of NMN and NR. Cell Metabol.

[5] Yoshino J., Mills K.F., Yoon M.J., Imai S. (2011). Nicotinamide mononucleotide, a key NAD(+) intermediate, treats the pathophysiology of diet- and age-induced diabetes in mice. Cell Metabol.

[6] Diguet N., Trammell S.A.J., Tannous C., Deloux R., Piquereau J., Mougenot N., Gouge A., Gressette M., Manoury B., Blanc J., Breton M., Decaux J.-F., Lavery G.G., Baczkó I., Zoll J., Garnier A., Li Z., Brenner C., Mericskay M. (2018). Nicotinamide riboside preserves cardiac function in a mouse model of dilated cardiomyopathy. Circulation.

[7] Sambeat A., Ratajczak J., Joffraud M., Sanchez-Garcia J.L., Giner M.P., Valsesia A., Giroud-Gerbetant J., Valera-Alberni M., Cercillieux A., Boutant M., Kulkarni S.S., Moco S., Canto C. (2019). Endogenous nicotinamide riboside metabolism protects against diet-induced liver damage. Nat Commun.

[8] Canner P.L., Furberg C.D., Terrin M.L., McGovern M.E. (2005). Benefits of niacin by glycemic status in patients with healed myocardial infarction (from the Coronary Drug Project). Am J Cardiol.

[9] Chu X., Raju R.P. (2022). Regulation of NAD(+) metabolism in aging and disease. Metabolism.

[10] Rajman L., Chwalek K., Sinclair D.A. (2018). Therapeutic potential of NAD-boosting molecules: the in vivo evidence. Cell Metabol.

[11] Nikiforov A., Kulikova V., Ziegler M. (2015). The human NAD metabolome: functions, metabolism and compartmentalization. Crit Rev Biochem Mol Biol.

[12] Rajman L., Chwalek K., Sinclair D.A. (2018). Therapeutic potential of NAD-boosting molecules: the in vivo evidence. Cell Metabol.

[13] Belenky P., Bogan K.L., Brenner C. (2007). NAD+ metabolism in health and disease. Trends Biochem Sci.

[14] Yang Y., Sauve A.A. (2016). NAD(+) metabolism: bioenergetics, signaling and manipulation for therapy. Biochim Biophys Acta.

[15] Sauve A.A. (2008). NAD+ and vitamin B3: from metabolism to therapies. J Pharmacol Exp Therapeut.

[16] Katsyuba E., Auwerx J. (2017). Modulating NAD(+) metabolism, from bench to bedside. EMBO J.

[17] Auger C., Vinaik R., Appanna V.D., Jeschke M.G. (2021). Beyond mitochondria: alternative energy-producing pathways from all strata of life. Metabolism.

[18] Ray Chaudhuri A., Nussenzweig A. (2017). The multifaceted roles of PARP1 in DNA repair and chromatin remodelling. Nat Rev Mol Cell Biol.

[19] Bogan K.L., Brenner C. (2008). Nicotinic acid, nicotinamide, and nicotinamide riboside: a molecular evaluation of NAD+ precursor vitamins in human nutrition. Annu Rev Nutr.

[20] Grozio A., Mills K.F., Yoshino J., Bruzzone S., Sociali G., Tokizane K., Lei H.C., Cunningham R., Sasaki Y., Migaud M.E., Imai S.-I. (2019). Slc12a8 is a nicotinamide mononucleotide transporter. Nat Metab.

[21] Ratajczak J., Joffraud M., Trammell S.A.J., Ras R., Canela N., Boutant M., Kulkarni S.S., Rodrigues M., Redpath P., Migaud M.E., Auwerx J., Yanes O., Brenner C., Cantó C. (2016). NRK1 controls nicotinamide mononucleotide and nicotinamide riboside metabolism in mammalian cells. Nat Commun.

[22] Ralto K.M., Rhee E.P., Parikh S.M. (2020). NAD(+) homeostasis in renal health and disease. Nat Rev Nephrol.

[23] Menzel S., Schwarz N., Haag F., Koch-Nolte F. (2018). Nanobody-based biologics for modulating purinergic signaling in inflammation and immunity. Front Pharmacol.

[24] Katsyuba E., Romani M., Hofer D., Auwerx J. (2020). NAD(+) homeostasis in health and disease. Nat Metab.

[25] Strømland Ø., Diab J., Ferrario E., Sverkeli L.J., Ziegler M. (2021). The balance between NAD(+) biosynthesis and consumption in ageing. Mech Ageing Dev.

[26] Liu L., Su X., Quinn W.J., Hui S., Krukenberg K., Frederick D.W., Redpath P., Zhan L., Chellappa K., White E., Migaud M., Mitchison T.J., Baur J.A., Rabinowitz J.D. (2018). Quantitative analysis of NAD synthesis-breakdown fluxes. Cell Metabol.

[27] Carrico C., Meyer J.G., He W., Gibson B.W., Verdin E. (2018). The mitochondrial acylome emerges: proteomics, regulation by sirtuins, and metabolic and disease implications. Cell Metabol.

[28] Rodriguez-Miguelez P., Looney J., Thomas J., Harshfield G., Pollock J.S., Harris R.A. (2020). Sirt1 during childhood is associated with microvascular function later in life. Am J Physiol Heart Circ Physiol.

[29] Lipphardt M., Song J.W., Ratliff B.B., Dihazi H., Müller G.A., Goligorsky M.S. (2018). Endothelial dysfunction is a superinducer of syndecan-4: fibrogenic role of its ectodomain. Am J Physiol Heart Circ Physiol.

[30] Bai P., Cantó C. (2012). The role of PARP-1 and PARP-2 enzymes in metabolic regulation and disease. Cell Metabol.

[31] Oliver A.W., Amé J.-C., Roe S.M., Good V., de Murcia G., Pearl L.H. (2004). Crystal structure of the catalytic fragment of murine poly(ADP-ribose) polymerase-2. Nucleic Acids Res.

[32] Boehler C., Gauthier L.R., Mortusewicz O., Biard D.S., Saliou J.-M., Bresson A., Sanglier-Cianferani S., Smith S., Schreiber V., Boussin F., Dantzer F. (2011). Poly(ADP-ribose) polymerase 3 (PARP3), a newcomer in cellular response to DNA damage and mitotic progression. Proc Natl Acad Sci USA.

[33] Zong W., Gong Y., Sun W., Li T., Wang Z.-Q. (2022). PARP1: liaison of chromatin remodeling and transcription. Cancers.

[34] Palazzo L., Suskiewicz M.J., Ahel I. (2021). Serine ADP-ribosylation in DNA-damage response regulation. Curr Opin Genet {\&} Dev.

[35] Vida A., Márton J., Mikó E., Bai P. (2017). Metabolic roles of poly(ADP-ribose) polymerases. Semin Cell {\&} Dev Biol.

[36] Pacher P., Szabo C. (2008). Role of the peroxynitrite-poly(ADP-ribose) polymerase pathway in human disease. Am J Pathol.

[37] Bai P., Cantó C., Oudart H., Brunyánszki A., Cen Y., Thomas C., Yamamoto H., Huber A., Kiss B., Houtkooper R.H., Schoonjans K., Schreiber V., Sauve A.A., Menissier-de Murcia J., Auwerx J. (2011). PARP-1 inhibition increases mitochondrial metabolism through SIRT1 activation. Cell Metab.

[38] Pirinen E., Cantó C., Jo Y.S., Morato L., Zhang H., Menzies K.J., Williams E.G., Mouchiroud L., Moullan N., Hagberg C., Li W., Timmers S., Imhof R., Verbeek J., Pujol A., van Loon B., Viscomi C., Zeviani M., Schrauwen P., Sauve A.A., Schoonjans K., Auwerx J. (2014). Pharmacological Inhibition of poly(ADP-ribose) polymerases improves fitness and mitochondrial function in skeletal muscle. Cell Metab.

[39] Graeff R., Liu Q., Kriksunov I.A., Hao Q., Lee H.C. (2006). Acidic residues at the active sites of CD38 and ADP-ribosyl cyclase determine nicotinic acid adenine dinucleotide phosphate (NAADP) synthesis and hydrolysis activities. J Biol Chem.

[40] Torti M., Bertoni A., Canobbio I., Sinigaglia F., Balduini C. (1999). Hydrolysis of NADP+ by platelet CD38 in the absence of synthesis and degradation of cyclic ADP-ribose 2’-phosphate. FEBS Lett.

[41] Camacho-Pereira J., Tarragó M.G., Chini C.C.S., Nin V., Escande C., Warner G.M., Puranik A.S., Schoon R.A., Reid J.M., Galina A., Chini E.N. (2016). CD38 dictates age-related NAD decline and mitochondrial dysfunction through an SIRT3-dependent mechanism. Cell Metab.

[42] Aomatsu E., Takahashi N., Sawada S., Okubo N., Hasegawa T., Taira M., Miura H., Ishisaki A., Chosa N. (2014). Novel SCRG1/BST1 axis regulates self-renewal, migration, and osteogenic differentiation potential in mesenchymal stem cells. Sci Rep.

[43] Preugschat F., Carter L.H., Boros E.E., Porter D.J.T., Stewart E.L., Shewchuk L.M. (2014). A pre-steady state and steady state kinetic analysis of the N-ribosyl hydrolase activity of hCD157. Arch Biochem Biophys.

[44] Pacinella G., Ciaccio A.M., Tuttolomondo A. (2022). Endothelial dysfunction and chronic inflammation: the cornerstones of vascular alterations in age-related diseases. Int J Mol Sci.

[45] Begum M.K., Konja D., Singh S., Chlopicki S., Wang Y. (2021). Endothelial SIRT1 as a target for the prevention of arterial aging: promises and challenges. J Cardiovasc Pharmacol.

[46] Li Y., Huang T.T., Carlson E.J., Melov S., Ursell P.C., Olson J.L., Noble L.J., Yoshimura M.P., Berger C., Chan P.H., Wallace D.C., Epstein C.J. (1995). Dilated cardiomyopathy and neonatal lethality in mutant mice lacking manganese superoxide dismutase. Nat Genet.

[47] Ardanaz N., Yang X.-P., Cifuentes M.E., Haurani M.J., Jackson K.W., Liao T.-D., Carretero O.A., Pagano P.J. (2010). Lack of glutathione peroxidase 1 accelerates cardiac-specific hypertrophy and dysfunction in angiotensin II hypertension. Hypertens (Dallas, Tex 1979.

[48] Chen Z., Chua C.C., Gao J., Chua K.-W., Ho Y.-S., Hamdy R.C., Chua B.H.L. (2009). Prevention of ischemia/reperfusion-induced cardiac apoptosis and injury by melatonin is independent of glutathione peroxdiase 1. J Pineal Res.

[49] Hu C., Zhang H., Qiao Z., Wang Y., Zhang P., Yang D. (2018). Loss of thioredoxin 2 alters mitochondrial respiratory function and induces cardiomyocyte hypertrophy. Exp Cell Res.

[50] Huang Q., Zhou H.J., Zhang H., Huang Y., Hinojosa-Kirschenbaum F., Fan P., Yao L., Belardinelli L., Tellides G., Giordano F.J., Budas G.R., Min W. (2015). Thioredoxin-2 inhibits mitochondrial reactive oxygen species generation and apoptosis stress kinase-1 activity to maintain cardiac function. Circulation.

[51] Amorim J.A., Coppotelli G., Rolo A.P., Palmeira C.M., Ross J.M., Sinclair D.A. (2022). Mitochondrial and metabolic dysfunction in ageing and age-related diseases. Nat Rev Endocrinol.

[52] de Picciotto N.E., Gano L.B., Johnson L.C., Martens C.R., Sindler A.L., Mills K.F., Imai S.-I., Seals D.R. (2016). Nicotinamide mononucleotide supplementation reverses vascular dysfunction and oxidative stress with aging in mice. Aging Cell.

[53] Hong G., Zheng D., Zhang L., Ni R., Wang G., Fan G.-C., Lu Z., Peng T. (2018). Administration of nicotinamide riboside prevents oxidative stress and organ injury in sepsis. Free Radic Biol {\&} Med.

[54] Tarantini S., Valcarcel-Ares M.N., Toth P., Yabluchanskiy A., Tucsek Z., Kiss T., Hertelendy P., Kinter M., Ballabh P., Süle Z., Farkas E., Baur J.A., Sinclair D.A., Csiszar A., Ungvari Z. (2019). Nicotinamide mononucleotide (NMN) supplementation rescues cerebromicrovascular endothelial function and neurovascular coupling responses and improves cognitive function in aged mice. Redox Biol.

[55] Kiss T., Nyúl-Tóth Á., Balasubramanian P., Tarantini S., Ahire C., Yabluchanskiy A., Csipo T., Farkas E., Wren J.D., Garman L., Csiszar A., Ungvari Z. (2020). Nicotinamide mononucleotide (NMN) supplementation promotes neurovascular rejuvenation in aged mice: transcriptional footprint of SIRT1 activation, mitochondrial protection, anti-inflammatory, and anti-apoptotic effects. GeroScience.

[56] Testai L., Citi V., Martelli A., Brogi S., Calderone V. (2020). Role of hydrogen sulfide in cardiovascular ageing. Pharmacol Res.

[57] Zhang W., Huang Q., Zeng Z., Wu J., Zhang Y., Chen Z. (2017). Sirt1 inhibits oxidative stress in vascular endothelial cells. Oxid Med Cell Longev.

[58] Klimova N., Long A., Kristian T. (2019). Nicotinamide mononucleotide alters mitochondrial dynamics by SIRT3-dependent mechanism in male mice. J Neurosci Res.

[59] Kiss T., Giles C.B., Tarantini S., Yabluchanskiy A., Balasubramanian P., Gautam T., Csipo T., Nyúl-Tóth Á., Lipecz A., Szabo C., Farkas E., Wren J.D., Csiszar A., Ungvari Z. (2019). Nicotinamide mononucleotide (NMN) supplementation promotes anti-aging miRNA expression profile in the aorta of aged mice, predicting epigenetic rejuvenation and anti-atherogenic effects. GeroScience.

[60] Aksoy P., White T.A., Thompson M., Chini E.N. (2006). Regulation of intracellular levels of NAD: a novel role for CD38. Biochem Biophys Res Commun.

[61] Boslett J., Hemann C., Christofi F.L., Zweier J.L. (2018). Characterization of CD38 in the major cell types of the heart: endothelial cells highly express CD38 with activation by hypoxia-reoxygenation triggering NAD(P)H depletion. Am J Physiol Cell Physiol.

[62] Ferrucci L., Fabbri E. (2018). Inflammageing: chronic inflammation in ageing, cardiovascular disease, and frailty. Nat Rev Cardiol.

[63] Furman D., Campisi J., Verdin E., Carrera-Bastos P., Targ S., Franceschi C., Ferrucci L., Gilroy D.W., Fasano A., Miller G.W., Miller A.H., Mantovani A., Weyand C.M., Barzilai N., Goronzy J.J., Rando T.A., Effros R.B., Lucia A., Kleinstreuer N., Slavich G.M. (2019). Chronic inflammation in the etiology of disease across the life span. Nat Med.

[64] Covarrubias A.J., Kale A., Perrone R., Lopez-Dominguez J.A., Pisco A.O., Kasler H.G., Schmidt M.S., Heckenbach I., Kwok R., Wiley C.D., Wong H.-S., Gibbs E., Iyer S.S., Basisty N., Wu Q., Kim I.-J., Silva E., Vitangcol K., Shin K.-O., Lee Y.-M., Riley R., Ben-Sahra I., Ott M., Schilling B., Scheibye-Knudsen M., Ishihara K., Quake S.R., Newman J., Brenner C., Campisi J., Verdin E. (2020). Senescent cells promote tissue NAD(+) decline during ageing via the activation of CD38(+) macrophages. Nat Metab.

[65] Amici S.A., Young N.A., Narvaez-Miranda J., Jablonski K.A., Arcos J., Rosas L., Papenfuss T.L., Torrelles J.B., Jarjour W.N., Guerau-de-Arellano M. (2018). CD38 is robustly induced in human macrophages and monocytes in inflammatory conditions. Front Immunol.

[66] Polzonetti V., Carpi F.M., Micozzi D., Pucciarelli S., Vincenzetti S., Napolioni V. (2012). Population variability in CD38 activity: correlation with age and significant effect of TNF-α -308G>A and CD38 184C>G SNPs. Mol Genet Metab.

[67] Weiss R., Schilling E., Grahnert A., Kölling V., Dorow J., Ceglarek U., Sack U., Hauschildt S. (2015). Nicotinamide: a vitamin able to shift macrophage differentiation toward macrophages with restricted inflammatory features. Innate Immun.

[68] Mitchell S.J., Bernier M., Aon M.A., Cortassa S., Kim E.Y., Fang E.F., Palacios H.H., Ali A., Navas-Enamorado I., Di Francesco A., Kaiser T.A., Waltz T.B., Zhang N., Ellis J.L., Elliott P.J., Frederick D.W., Bohr V.A., Schmidt M.S., Brenner C., Sinclair D.A., Sauve A.A., Baur J.A., de Cabo R. (2018). Nicotinamide improves aspects of healthspan, but not lifespan, in mice. Cell Metab.

[69] Kong D., Li J., Shen Y., Liu G., Zuo S., Tao B., Ji Y., Lu A., Lazarus M., Breyer R.M., Yu Y. (2017). Niacin promotes cardiac healing after myocardial infarction through activation of the myeloid prostaglandin D(2) receptor subtype 1. J Pharmacol Exp Ther.

[70] Mateuszuk Ł., Campagna R., Kutryb-Zając B., Kuś K., Słominska E.M., Smolenski R.T., Chlopicki S. (2020). Reversal of endothelial dysfunction by nicotinamide mononucleotide via extracellular conversion to nicotinamide riboside. Biochem Pharmacol.

[71] Abdellatif M., Sedej S., Carmona-Gutierrez D., Madeo F., Kroemer G. (2018). Autophagy in cardiovascular aging. Circ Res.

[72] Osonoi Y., Mita T., Azuma K., Nakajima K., Masuyama A., Goto H., Nishida Y., Miyatsuka T., Fujitani Y., Koike M., Mitsumata M., Watada H. (2018). Defective autophagy in vascular smooth muscle cells enhances cell death and atherosclerosis. Autophagy.

[73] LaRocca T.J., Henson G.D., Thorburn A., Sindler A.L., Pierce G.L., Seals D.R. (2012). Translational evidence that impaired autophagy contributes to arterial ageing. J Physiol.

[74] Zhang Y.-J., Zhang M., Zhao X., Shi K., Ye M., Tian J., Guan S., Ying W., Qu X. (2020). NAD(+) administration decreases microvascular damage following cardiac ischemia/reperfusion by restoring autophagic flux. Basic Res Cardiol.

[75] Lee I.H., Cao L., Mostoslavsky R., Lombard D.B., Liu J., Bruns N.E., Tsokos M., Alt F.W., Finkel T. (2008). A role for the NAD-dependent deacetylase Sirt1 in the regulation of autophagy. Proc Natl Acad Sci U S A.

[76] Wolf D., Ley K. (2019). Immunity and inflammation in atherosclerosis. Circ Res.

[77] Mallat Z. (2014). Macrophages. Arterioscler Thromb Vasc Biol.

[78] Feil S., Fehrenbacher B., Lukowski R., Essmann F., Schulze-Osthoff K., Schaller M., Feil R. (2014). Transdifferentiation of vascular smooth muscle cells to macrophage-like cells during atherogenesis. Circ Res.

[79] Li X., Zhang S., Blander G., Tse J.G., Krieger M., Guarente L. (2007). SIRT1 deacetylates and positively regulates the nuclear receptor LXR. Mol Cell.

[80] Sosnowska B., Mazidi M., Penson P., Gluba-Brzózka A., Rysz J., Banach M. (2017). The sirtuin family members SIRT1, SIRT3 and SIRT6: their role in vascular biology and atherogenesis. Atherosclerosis.

[81] Méndez-Lara K.A., Letelier N., Farré N., Diarte-Añazco E.M.G., Nieto-Nicolau N., Rodríguez-Millán E., Santos D., Pallarès V., Escolà-Gil J.C., Vázquez Del Olmo T., Lerma E., Camacho M., Casaroli-Marano R.P., Valledor A.F., Blanco-Vaca F., Julve J. (2020). Nicotinamide prevents apolipoprotein B-containing lipoprotein oxidation, inflammation and atherosclerosis in apolipoprotein E-deficient mice. Antioxidants (Basel, Switzerland).

[82] Domagala T.B., Szeffler A., Dobrucki L.W., Dropinski J., Polanski S., Leszczynska-Wiloch M., Kotula-Horowitz K., Wojciechowski J., Wojnowski L., Szczeklik A., Kalinowski L. (2012). Nitric oxide production and endothelium-dependent vasorelaxation ameliorated by N1-methylnicotinamide in human blood vessels. Hypertens (Dallas, Tex 1979).

[83] Mateuszuk L., Jasztal A., Maslak E., Gasior-Glogowska M., Baranska M., Sitek B., Kostogrys R., Zakrzewska A., Kij A., Walczak M., Chlopicki S. (2016). Antiatherosclerotic effects of 1-methylnicotinamide in apolipoprotein E/low-density lipoprotein receptor-deficient mice: a comparison with nicotinic acid. J Pharmacol Exp Ther.

[84] Jiang N., Wang M., Song J., Liu Y., Chen H., Mu D., Xia M. (2016). N-methylnicotinamide protects against endothelial dysfunction and attenuates atherogenesis in apolipoprotein E-deficient mice. Mol Nutr {\&} Food Res.

[85] Li S., Wang C., Li K., Li L., Tian M., Xie J., Yang M., Jia Y., He J., Gao L., Boden G., Liu H., Yang G. (2016). NAMPT knockdown attenuates atherosclerosis and promotes reverse cholesterol transport in ApoE KO mice with high-fat-induced insulin resistance. Sci Rep.

[86] Bermudez B., Dahl T.B., Medina I., Groeneweg M., Holm S., Montserrat-de la Paz S., Rousch M., Otten J., Herias V., Varela L.M., Ranheim T., Yndestad A., Ortega-Gomez A., Abia R., Nagy L., Aukrust P., Muriana F.J.G., Halvorsen B., Biessen E.A.L. (2017). Leukocyte overexpression of intracellular NAMPT attenuates atherosclerosis by regulating PPAR$γ$-Dependent monocyte differentiation and function. Arterioscler Thromb Vasc Biol.

[87] Kong Y.-Y., Li G.-Q., Zhang W.-J., Hua X., Zhou C.-C., Xu T.-Y., Li Z.-Y., Wang P., Miao C.-Y. (2019). Nicotinamide phosphoribosyltransferase aggravates inflammation and promotes atherosclerosis in ApoE knockout mice. Acta Pharmacol Sin.

[88] Zheng C., Han J., Xia W., Shi S., Liu J., Ying W. (2012). NAD(+) administration decreases ischemic brain damage partially by blocking autophagy in a mouse model of brain ischemia. Neurosci Lett.

[89] Xu S., Bai P., Little P.J., Liu P. (2014). Poly(ADP-ribose) polymerase 1 (PARP1) in atherosclerosis: from molecular mechanisms to therapeutic implications. Med Res Rev.

[90] Oumouna-Benachour K., Hans C.P., Suzuki Y., Naura A., Datta R., Belmadani S., Fallon K., Woods C., Boulares A.H. (2007). Poly(ADP-ribose) polymerase inhibition reduces atherosclerotic plaque size and promotes factors of plaque stability in apolipoprotein E-deficient mice: effects on macrophage recruitment, nuclear factor-kappaB nuclear translocation, and foam cell death. Circulation.

[91] von Lukowicz T., Hassa P.O., Lohmann C., Borén J., Braunersreuther V., Mach F., Odermatt B., Gersbach M., Camici G.G., Stähli B.E., Tanner F.C., Hottiger M.O., Lüscher T.F., Matter C.M. (2008). PARP1 is required for adhesion molecule expression in atherogenesis. Cardiovasc Res.

[92] Hans C.P., Zerfaoui M., Naura A.S., Troxclair D., Strong J.P., Matrougui K., Boulares A.H. (2009). Thieno[2,3-c]isoquinolin-5-one, a potent poly(ADP-ribose) polymerase inhibitor, promotes atherosclerotic plaque regression in high-fat diet-fed apolipoprotein E-deficient mice: effects on inflammatory markers and lipid content. J Pharmacol Exp Ther.

[93] Zha S., Wang F., Li Z., Ma Z., Yang L., Liu F. (2019). PJ34, a PARP1 inhibitor, promotes endothelial repair in a rabbit model of high fat diet-induced atherosclerosis. Cell Cycle.

[94] V Sukoyan G., Kavadze I.K. (2008). Effect of nadcin on energy supply system and apoptosis in ischemia-reperfusion injury to the myocardium. Bull Exp Biol Med.

[95] V Sukoyan G., Andriadze N.A., Guchua E.I., V Karsanov N. (2005). Effect of NAD on recovery of adenine nucleotide pool, phosphorylation potential, and stimulation of apoptosis during late period of reperfusion damage to myocardium. Bull Exp Biol Med.

[96] Liaudet L., Yang Z., Al-Affar E.B., Szabó C. (2001). Myocardial ischemic preconditioning in rodents is dependent on poly (ADP-ribose) synthetase. Mol Med.

[97] Reyes L.A., Boslett J., Varadharaj S., De Pascali F., Hemann C., Druhan L.J., Ambrosio G., El-Mahdy M., Zweier J.L. (2015). Depletion of NADP(H) due to CD38 activation triggers endothelial dysfunction in the postischemic heart. Proc Natl Acad Sci U S A.

[98] Zhang Y., Wang B., Fu X., Guan S., Han W., Zhang J., Gan Q., Fang W., Ying W., Qu X. (2016). Exogenous NAD(+) administration significantly protects against myocardial ischemia/reperfusion injury in rat model. Am J Transl Res.

[99] Zhang Y., Sun L., Xuan L., Pan Z., Li K., Liu S., Huang Y., Zhao X., Huang L., Wang Z., Hou Y., Li J., Tian Y., Yu J., Han H., Liu Y., Gao F., Zhang Y., Wang S., Du Z., Lu Y., Yang B. (2016). Reciprocal changes of circulating long non-coding RNAs ZFAS1 and CDR1AS predict acute myocardial infarction. Sci Rep.

[100] Liu Y., Wang J., Zhao X., Li W., Liu Y., Li X., Zhao D., Yu J., Ji H., Shao B., Li Z., Wang J., Yang Y., Hao Y., Wu Y., Yuan Y., Du Z. (2023). CDR1as promotes arrhythmias in myocardial infarction via targeting the NAMPT-NAD(+) pathway. Biomed {\&} Pharmacother = Biomed {\&} Pharmacother.

[101] Hsu C.-P., Oka S., Shao D., Hariharan N., Sadoshima J. (2009). Nicotinamide phosphoribosyltransferase regulates cell survival through NAD+ synthesis in cardiac myocytes. Circ Res.

[102] Zhai X., Han W., Wang M., Guan S., Qu X. (2019). Exogenous supplemental NAD+ protect myocardium against myocardial ischemic/reperfusion injury in swine model. Am J Transl Res.

[103] Sukhodub A., Du Q., Jovanović S., Jovanović A. (2010). Nicotinamide-rich diet protects the heart against ischaemia-reperfusion in mice: a crucial role for cardiac SUR2A. Pharmacol Res.

[104] Hosseini L., Vafaee M.S., Badalzadeh R. (2020). Melatonin and nicotinamide mononucleotide attenuate myocardial ischemia/reperfusion injury via modulation of mitochondrial function and hemodynamic parameters in aged rats. J Cardiovasc Pharmacol Ther.

[105] Gan L., Liu D., Liu J., Chen E., Chen C., Liu L., Hu H., Guan X., Ma W., Zhang Y., He Y., Liu B., Tang S., Jiang W., Xue J., Xin H. (2021). CD38 deficiency alleviates Ang II-induced vascular remodeling by inhibiting small extracellular vesicle-mediated vascular smooth muscle cell senescence in mice. Signal Transduct Target Ther.

[106] Zhang H., Ryu D., Wu Y., Gariani K., Wang X., Luan P., D'Amico D., Ropelle E.R., Lutolf M.P., Aebersold R., Schoonjans K., Menzies K.J., Auwerx J. (2016). NAD^+^ repletion improves mitochondrial and stem cell function and enhances life span in mice. Science.

[107] Chini C.C.S., Peclat T.R., Warner G.M., Kashyap S., Espindola-Netto J.M., de Oliveira G.C., Gomez L.S., Hogan K.A., Tarragó M.G., Puranik A.S., Agorrody G., Thompson K.L., Dang K., Clarke S., Childs B.G., Kanamori K.S., Witte M.A., Vidal P., Kirkland A.L., De Cecco M., Chellappa K., McReynolds M.R., Jankowski C., Tchkonia T., Kirkland J.L., Sedivy J.M., van Deursen J.M., Baker D.J., van Schooten W., Rabinowitz J.D., Baur J.A., Chini E.N. (2020). CD38 ecto-enzyme in immune cells is induced during aging and regulates NAD(+) and NMN levels. Nat Metab.

[108] Gomes A.P., Price N.L., Ling A.J.Y., Moslehi J.J., Montgomery M.K., Rajman L., White J.P., Teodoro J.S., Wrann C.D., Hubbard B.P., Mercken E.M., Palmeira C.M., de Cabo R., Rolo A.P., Turner N., Bell E.L., Sinclair D.A. (2013). Declining NAD(+) induces a pseudohypoxic state disrupting nuclear-mitochondrial communication during aging. Cell.

[109] McReynolds M.R., Chellappa K., Baur J.A. (2020). Age-related NAD(+) decline. Exp Gerontol.

[110] Mouchiroud L., Houtkooper R.H., Moullan N., Katsyuba E., Ryu D., Cantó C., Mottis A., Jo Y.-S., Viswanathan M., Schoonjans K., Guarente L., Auwerx J. (2013). The NAD(+)/Sirtuin pathway modulates longevity through activation of mitochondrial UPR and FOXO signaling. Cell.

[111] Yoshida M., Satoh A., Lin J.B., Mills K.F., Sasaki Y., Rensing N., Wong M., Apte R.S., Imai S.-I. (2019). Extracellular vesicle-contained eNAMPT delays aging and extends lifespan in mice. Cell Metab.

[112] López-Otín C., Galluzzi L., Freije J.M.P., Madeo F., Kroemer G. (2016). Metabolic control of longevity. Cell.

[113] López-Otín C., Kroemer G. (2021). Hallmarks of health. Cell.

[114] Leonard S.S., Xia C., Jiang B.-H., Stinefelt B., Klandorf H., Harris G.K., Shi X. (2003). Resveratrol scavenges reactive oxygen species and effects radical-induced cellular responses. Biochem Biophys Res Commun.

[115] Truong V.-L., Jun M., Jeong W.-S. (2018). Role of resveratrol in regulation of cellular defense systems against oxidative stress. Biofactors.

[116] Carter L.G., D'Orazio J.A., Pearson K.J. (2014). Resveratrol and cancer: focus on in vivo evidence. Endocr Relat Cancer.

[117] Shrotriya S., Agarwal R., Sclafani R.A. (2015). A perspective on chemoprevention by resveratrol in head and neck squamous cell carcinoma. Adv Exp Med Biol.

[118] Xu Q., Zong L., Chen X., Jiang Z., Nan L., Li J., Duan W., Lei J., Zhang L., Ma J., Li X., Wang Z., Wu Z., Ma Q., Ma Z. (2015). Resveratrol in the treatment of pancreatic cancer. Ann N Y Acad Sci.

[119] Bhullar K.S., Hubbard B.P. (2015). Lifespan and healthspan extension by resveratrol. Biochim Biophys Acta.

[120] Li Y.-R., Li S., Lin C.-C. (2018). Effect of resveratrol and pterostilbene on aging and longevity. Biofactors.

[121] Howitz K.T., Bitterman K.J., Cohen H.Y., Lamming D.W., Lavu S., Wood J.G., Zipkin R.E., Chung P., Kisielewski A., Zhang L.-L., Scherer B., Sinclair D.A. (2003). Small molecule activators of sirtuins extend Saccharomyces cerevisiae lifespan. Nature.

[122] Wood J.G., Rogina B., Lavu S., Howitz K., Helfand S.L., Tatar M., Sinclair D. (2004). Sirtuin activators mimic caloric restriction and delay ageing in metazoans. Nature.

[123] Baur J.A., Pearson K.J., Price N.L., Jamieson H.A., Lerin C., Kalra A., V Prabhu V., Allard J.S., Lopez-Lluch G., Lewis K., Pistell P.J., Poosala S., Becker K.G., Boss O., Gwinn D., Wang M., Ramaswamy S., Fishbein K.W., Spencer R.G., Lakatta E.G., Le Couteur D., Shaw R.J., Navas P., Puigserver P., Ingram D.K., de Cabo R., Sinclair D.A. (2006). Resveratrol improves health and survival of mice on a high-calorie diet. Nature.

[124] Lagouge M., Argmann C., Gerhart-Hines Z., Meziane H., Lerin C., Daussin F., Messadeq N., Milne J., Lambert P., Elliott P., Geny B., Laakso M., Puigserver P., Auwerx J. (2006). Resveratrol improves mitochondrial function and protects against metabolic disease by activating SIRT1 and PGC-1 alpha. Cell.

[125] Barger J.L., Kayo T., Vann J.M., Arias E.B., Wang J., Hacker T.A., Wang Y., Raederstorff D., Morrow J.D., Leeuwenburgh C., Allison D.B., Saupe K.W., Cartee G.D., Weindruch R., Prolla T.A. (2008). A low dose of dietary resveratrol partially mimics caloric restriction and retards aging parameters in mice. PLoS One.

[126] Cantó C., Gerhart-Hines Z., Feige J.N., Lagouge M., Noriega L., Milne J.C., Elliott P.J., Puigserver P., Auwerx J. (2009). AMPK regulates energy expenditure by modulating NAD+ metabolism and SIRT1 activity. Nature.

[127] Fulco M., Cen Y., Zhao P., Hoffman E.P., McBurney M.W., Sauve A.A., Sartorelli V. (2008). Glucose restriction inhibits skeletal myoblast differentiation by activating SIRT1 through AMPK-mediated regulation of Nampt. Dev Cell.

[128] Hou X., Xu S., Maitland-Toolan K.A., Sato K., Jiang B., Ido Y., Lan F., Walsh K., Wierzbicki M., Verbeuren T.J., Cohen R.A., Zang M. (2008). SIRT1 regulates hepatocyte lipid metabolism through activating AMP-activated protein kinase. J Biol Chem.

[129] Ivanov V.N., Partridge M.A., Johnson G.E., Huang S.X.L., Zhou H., Hei T.K. (2008). Resveratrol sensitizes melanomas to TRAIL through modulation of antiapoptotic gene expression. Exp Cell Res.

[130] Lan F., Cacicedo J.M., Ruderman N., Ido Y. (2008). SIRT1 modulation of the acetylation status, cytosolic localization, and activity of LKB1. Possible role in AMP-activated protein kinase activation. J Biol Chem.

[131] Price N.L., Gomes A.P., Ling A.J.Y., V Duarte F., Martin-Montalvo A., North B.J., Agarwal B., Ye L., Ramadori G., Teodoro J.S., Hubbard B.P., Varela A.T., Davis J.G., Varamini B., Hafner A., Moaddel R., Rolo A.P., Coppari R., Palmeira C.M., de Cabo R., Baur J.A., Sinclair D.A. (2012). SIRT1 is required for AMPK activation and the beneficial effects of resveratrol on mitochondrial function. Cell Metab.

[132] Howitz K.T., Sinclair D.A. (2008). Xenohormesis: sensing the chemical cues of other species. Cell.

[133] Timmers S., Konings E., Bilet L., Houtkooper R.H., van de Weijer T., Goossens G.H., Hoeks J., van der Krieken S., Ryu D., Kersten S., Moonen-Kornips E., Hesselink M.K.C., Kunz I., Schrauwen-Hinderling V.B., Blaak E., Auwerx J., Schrauwen P. (2011). Calorie restriction-like effects of 30 days of resveratrol supplementation on energy metabolism and metabolic profile in obese humans. Cell Metab.

[134] Poulsen M.M., Vestergaard P.F., Clasen B.F., Radko Y., Christensen L.P., Stødkilde-Jørgensen H., Møller N., Jessen N., Pedersen S.B., Jørgensen J.O.L. (2013). High-dose resveratrol supplementation in obese men: an investigator-initiated, randomized, placebo-controlled clinical trial of substrate metabolism, insulin sensitivity, and body composition. Diabetes.

[135] Uddin M.J., Farjana M., Moni A., Hossain K.S., Hannan M.A., Ha H. (2021). Prospective pharmacological potential of resveratrol in delaying kidney aging. Int J Mol Sci.

[136] Kim E.N., Kim M.Y., Lim J.H., Kim Y., Shin S.J., Park C.W., Kim Y.-S., Chang Y.S., Yoon H.E., Choi B.S. (2018). The protective effect of resveratrol on vascular aging by modulation of the renin-angiotensin system. Atherosclerosis.

[137] Caldeira C.A., Santos M.A., Araújo G.R., Lara R.C., Franco F.N., Chaves M.M. (2021). Resveratrol: change of SIRT 1 and AMPK signaling pattern during the aging process. Exp Gerontol.

[138] Martens C.R., Denman B.A., Mazzo M.R., Armstrong M.L., Reisdorph N., McQueen M.B., Chonchol M., Seals D.R. (2018). Chronic nicotinamide riboside supplementation is well-tolerated and elevates NAD(+) in healthy middle-aged and older adults. Nat Commun.

[139] Airhart S.E., Shireman L.M., Risler L.J., Anderson G.D., Nagana Gowda G.A., Raftery D., Tian R., Shen D.D., O'Brien K.D. (2017). An open-label, non-randomized study of the pharmacokinetics of the nutritional supplement nicotinamide riboside (NR) and its effects on blood NAD+ levels in healthy volunteers. PLoS One.

[140] Trammell S.A.J., Schmidt M.S., Weidemann B.J., Redpath P., Jaksch F., Dellinger R.W., Li Z., Abel E.D., Migaud M.E., Brenner C. (2016). Nicotinamide riboside is uniquely and orally bioavailable in mice and humans. Nat Commun.

[141] Zhou B., Wang D.D.-H., Qiu Y., Airhart S., Liu Y., Stempien-Otero A., O'Brien K.D., Tian R. (2020). Boosting NAD level suppresses inflammatory activation of PBMCs in heart failure. J Clin Invest.

[142] Vreones M., Mustapic M., Moaddel R., Pucha K.A., Lovett J., Seals D.R., Kapogiannis D., Martens C.R. (2023). Oral nicotinamide riboside raises NAD+ and lowers biomarkers of neurodegenerative pathology in plasma extracellular vesicles enriched for neuronal origin. Aging Cell.

[143] Freeberg K.A., Craighead D.H., Martens C.R., You Z., Chonchol M., Seals D.R. (2022). Nicotinamide riboside supplementation for treating elevated systolic blood pressure and arterial stiffness in midlife and older adults. Front Cardiovasc Med.

[144] Elhassan Y.S., Kluckova K., Fletcher R.S., Schmidt M.S., Garten A., Doig C.L., Cartwright D.M., Oakey L., V Burley C., Jenkinson N., Wilson M., Lucas S.J.E., Akerman I., Seabright A., Lai Y.-C., Tennant D.A., Nightingale P., Wallis G.A., Manolopoulos K.N., Brenner C., Philp A., Lavery G.G. (2019). Nicotinamide riboside augments the aged human skeletal muscle NAD(+) metabolome and induces transcriptomic and anti-inflammatory signatures. Cell Rep.

[145] Wang D.D., Airhart S.E., Zhou B., Shireman L.M., Jiang S., Melendez Rodriguez C., Kirkpatrick J.N., Shen D.D., Tian R., O'Brien K.D. (2022). Safety and tolerability of nicotinamide riboside in heart failure with reduced ejection fraction. JACC Basic to Transl Sci.

[146] Romani M., Hofer D.C., Katsyuba E., Auwerx J. (2019). Niacin: an old lipid drug in a new NAD(+) dress. J Lipid Res.

[147] Misner D.L., Kauss M.A., Singh J., Uppal H., Bruening-Wright A., Liederer B.M., Lin T., McCray B., La N., Nguyen T., Sampath D., Dragovich P.S., O'Brien T., Zabka T.S. (2017). Cardiotoxicity associated with nicotinamide phosphoribosyltransferase inhibitors in rodents and in rat and human-derived cells lines. Cardiovasc Toxicol.

[148] Rahman J.E., Helou E.F., Gelzer-Bell R., Thompson R.E., Kuo C., Rodriguez E.R., Hare J.M., Baughman K.L., Kasper E.K. (2004). Noninvasive diagnosis of biopsy-proven cardiac amyloidosis. J Am Coll Cardiol.

[149] Frustaci A., Verardo R., Caldarulo M., Acconcia M.C., Russo M.A., Chimenti C. (2007). Myocarditis in hypertrophic cardiomyopathy patients presenting acute clinical deterioration. Eur Heart J.

[150] Clerkin K.J., Fried J.A., Raikhelkar J., Sayer G., Griffin J.M., Masoumi A., Jain S.S., Burkhoff D., Kumaraiah D., Rabbani L., Schwartz A., Uriel N. (2020). COVID-19 and cardiovascular disease. Circulation.

[151] Badawy A.A.-B. (2020). Immunotherapy of COVID-19 with poly (ADP-ribose) polymerase inhibitors: starting with nicotinamide. Biosci Rep.

[152] Miller R., Wentzel A.R., Richards G.A. (2020). COVID-19: NAD(+) deficiency may predispose the aged, obese and type 2 diabetics to mortality through its effect on SIRT1 activity. Med Hypotheses.

[153] Omran H.M., Almaliki M.S. (2020). Influence of NAD+ as an ageing-related immunomodulator on COVID 19 infection: a hypothesis. J Infect Public Health.

[154] Raines N.H., Ganatra S., Nissaisorakarn P., Pandit A., Morales A., Asnani A., Sadrolashrafi M., Maheshwari R., Patel R., Bang V., Shreyder K., Brar S., Singh A., Dani S.S., Knapp S., Poyan Mehr A., Brown R.S., Zeidel M.L., Bhargava R., Schlondorff J., Steinman T.I., Mukamal K.J., Parikh S.M. (2021). Niacinamide may Be associated with improved outcomes in COVID-19-related acute kidney injury: an observational study. Kidney360.

[155] Mills K.F., Yoshida S., Stein L.R., Grozio A., Kubota S., Sasaki Y., Redpath P., Migaud M.E., Apte R.S., Uchida K., Yoshino J., Imai S.-I. (2016). Long-term administration of nicotinamide mononucleotide mitigates age-associated physiological decline in mice. Cell Metab.

[156] Liao B., Zhao Y., Wang D., Zhang X., Hao X., Hu M. (2021). Nicotinamide mononucleotide supplementation enhances aerobic capacity in amateur runners: a randomized, double-blind study. J Int Soc Sports Nutr.

[157] Wen D.-T., Zheng L., Lu K., Hou W.-Q. (2021). Activation of cardiac Nmnat/NAD+/SIR2 pathways mediates endurance exercise resistance to lipotoxic cardiomyopathy in aging Drosophila. J Exp Biol.

[158] Irie J., Inagaki E., Fujita M., Nakaya H., Mitsuishi M., Yamaguchi S., Yamashita K., Shigaki S., Ono T., Yukioka H., Okano H., Nabeshima Y.-I., Imai S.-I., Yasui M., Tsubota K., Itoh H. (2020). Effect of oral administration of nicotinamide mononucleotide on clinical parameters and nicotinamide metabolite levels in healthy Japanese men. Endocr J.

